# A phase I study of a new polyamine biosynthesis inhibitor, SAM486A, in cancer patients with solid tumours

**DOI:** 10.1054/bjoc.2000.1305

**Published:** 2000-08-16

**Authors:** R Paridaens, D R A Uges, N Barbet, L Choi, M Seeghers, W T A van der Graaf, H J M Groen, H Dumez, I Van Buuren, F Muskiet, R Capdeville, A T van Oosterom, E G E de Vries

**Affiliations:** Universitair Ziekenhuis Gasthuisberg, Herestraat 49, Leuven, 3000, Belgium; Academisch Ziekenhuis Groningen, Groningen, EZ, 9713, The Netherlands; Novartis Pharma AG, Lichtstrasse 35, Basel, CH-4002, Switzerland; Novartis Pharmaceuticals Corporation, 59 Route 10, East Hanover, NJ, 07936, USA; Novartis Pharma, PB 241, Arnhem, LZ, 6800, the Netherlands

**Keywords:** polyamine, SAMDC, phase I, 5FU, neutropenia

## Abstract

Because tumour cell proliferation is highly dependent upon up-regulation of de-novo polyamine synthesis, inhibition of the polyamine synthesis pathway represents a potential target for anticancer therapy. SAM486A (CGP 48664) is a new inhibitor of the polyamine biosynthetic enzyme S-adenosylmethionine decarboxylase (SAMDC), more potent and specific than the first-generation SAMDC inhibitor methylglyoxal (bis) guanylhydrazone (MGBG). Preclinical testing confirmed promising antiproliferative activity. In this phase I study, SAM486A was given 4-weekly as a 120 h infusion. 39 adult cancer patients were enrolled with advanced/refractory disease not amenable to established treatments, PS ≤ 2, adequate marrow, liver, renal and cardiac function. Doses were escalated in 100% increments without toxicity in 24 pts from 3 mg m^–2^cycle^–1^up to 400 mg m^–2^cycle^–1^. At 550 and 700 mg m^–2^cycle^–1^reversible dose-limiting neutropenia occurred. Other toxicities included mild fatigue, nausea and vomiting. No objective remission was seen. Pharmakokinetic analysis showed a terminal half-life of approximately 2 days. AUC and Cmax were related to dose; neutropenia correlated with AUC. The recommended dose for further phase II studies on this schedule is 400 mg m^–2^cycle^–1^. © 2000 Cancer Research Campaign


*British Journal of Cancer* (2000) **83**(5), 594–601
© 2000 Cancer Research Campaign
doi: 10.1054/ bjoc.2000.1305, available online at http://www.idealibrary.com on IDEAL®

### A phase I study of a new polyamine biosynthesis inhibitor, SAM486A, in cancer patients with solid tumours

**R Paridaens[1], DRA Uges[2], N Barbet[3], L Choi[4], M Seeghers[1], WTA van der Graaf[2], HJM Groen[2], H Dumez[1], I Van Buuren[5], F Muskiet[2], R Capdeville[3], AT van Oosterom[1] and EGE de Vries[2]**

[1]Universitair Ziekenhuis Gasthuisberg, Herestraat 49, 3000 Leuven, Belgium; [2]Academisch Ziekenhuis Groningen, 9713 EZ Groningen, The Netherlands; [3]Novartis Pharma AG, Lichtstrasse 35, CH-4002 Basel, Switzerland: [4]Novartis Pharmaceuticals Corporation, 59 Route 10, East Hanover, NJ 07936, USA; [5]Novartis Pharma, PB 241, 6800 LZ Arnhem, the Netherlands

**Summary** Because tumour cell proliferation is highly dependent upon up-regulation of de-novo polyamine synthesis, inhibition of the polyamine synthesis pathway represents a potential target for anticancer therapy. SAM486A (CGP 48664) is a new inhibitor of the polyamine biosynthetic enzyme S-adenosylmethionine decarboxylase (SAMDC), more potent and specific than the first-generation SAMDC inhibitor methylglyoxal (bis) guanylhydrazone (MGBG). Preclinical testing confirmed promising antiproliferative activity. In this phase I study, SAM486A was given 4-weekly as a 120 h infusion. 39 adult cancer patients were enrolled with advanced/refractory disease not amenable to established treatments, PS ≤ 2, adequate marrow, liver, renal and cardiac function. Doses were escalated in 100% increments without toxicity in 24 pts from 3 mg $m^{-2}$ $cycle^{-1}$ up to 400 mg $m^{-2}$ $cycle^{-1}$. At 550 and 700 mg $m^{-2}$ $cycle^{-1}$ reversible dose-limiting neutropenia occurred. Other toxicities included mild fatigue, nausea and vomiting. No objective remission was seen. Pharmakokinetic analysis showed a terminal half-life of approximately 2 days. AUC and Cmax were related to dose; neutropenia correlated with AUC. The recommended dose for further phase II studies on this schedule is 400 mg $m^{-2}$ $cycle^{-1}$. © 2000 Cancer Research Campaign

**Keywords:** polyamine; SAMDC; phase I; 5FU; neutropenia

The synthesis of the diamine putrescine, and the polyamines spermidine and spermine, is of vital importance for cell proliferation. Due to their polycationic nature, polyamines interact with various macromolecules and influence multiple cellular functions. Growth stimuli strongly induce polyamine biosynthesis, which appears to be a prerequisite for cell proliferation. Tumour cells appear to have an altered polyamine homeostasis reflected by increased activity of biosynthetic enzymes and elevated polyamine pools (Porter et al, 1992). Hence, the two rate-limiting enzymes of polyamine biosynthesis, ornithine decarboxylase (ODC) (McCann and Pegg, 1992) and S-adenosylmethionine decarboxylase (SAMDC) (Pegg and McCann, 1992) have been selected as potential therapeutic targets for cancer chemotherapy. The polyamine biosynthesis pathway is depicted in Figure 1. Since the early 1960s, preclinical and clinical trials have been undertaken with the SAMDC inhibitor, methylglyoxal bis(guanylhydrazone) (MGBG) in cancer indications. Responses were noted in a broad spectrum of malignant diseases, including acute leukaemia, chronic myelogenous leukaemia, lymphoma, multiple myeloma, head and neck cancer, and oesophageal cancer. However, toxicity was marked and consisted of myelosuppression and mucositis, as well as mitochondrial toxicity (Warrell and Burchenal, 1983). Because of MGBG's unfavourable safety profile, new SAMDC inhibitors were sought with improved potency and specificity.

*Received 16 September 1999*
*Revised 8 March 2000*
*Accepted 13 April 2000*

*Correspondence to:* R Paridaens

SAM486A (CGP 48664) is the free base of 4-(aminoiminomethyl)-2,3-dihydro-1H-inden-1-one-diaminomethylenehydrazone whose salt with D,L-lactic acid was the formulation used for intravenous administration in this study. In vitro, this compound is a potent, selective inhibitor of SAMDC, with an $IC_{50}$ of 4.7 nM, being approximately 200-fold more active than MGBG (Regenass et al, 1994). It showed growth inhibitory activity against a variety of different tumour cell lines ($IC_{50}$ in the micromolar range), including multi-drug-resistant and highly metastatic variants (H Mett, unpublished data). In in vivo mice xenograft or orthotopic tumour experiments, doses of SAM486 ranging from 5–25 mg $kg^{-1}$ $day^{-1}$ given intraperitoneally inhibited tumour growth (Regenass et al, 1994; Dorhout et al, 1995; Gutman et al, 1995; Manni et al, 1995; Delworth et al, 1997; Svensson et al, 1997; Mi et al, 1998). Further xenograft experiments suggested that a daily schedule was associated with a better tumour growth control as compared to an intermittent schedule (data on file). Additive to synergistic effects both in vitro and in vivo were observed when SAM486A was combined with 5-fluorouracil (5FU) and with cisplatin (Gschaidmeier et al, 1999, O'Reilly et al, 2000). Tumours of treated L-1210 leukaemia-bearing animals showed decreased SAMDC activity and elevated putrescine pools (Dorhout et al, 1995). In toxicology studies, short-term single or repeat dose treatment of rats and dogs with bolus injections of high doses of SAM486A induced acute cardiovascular and respiratory symptoms, described as hyperaemia, tachycardia, cyanosis, reduced body temperature, and dyspnoea, gasping, deep respiration, respectively. After long-term treatment, heterogenous ECG alterations in dogs were observed, together with morphological changes in liver and heart. Clearance of SAM486A from plasma of

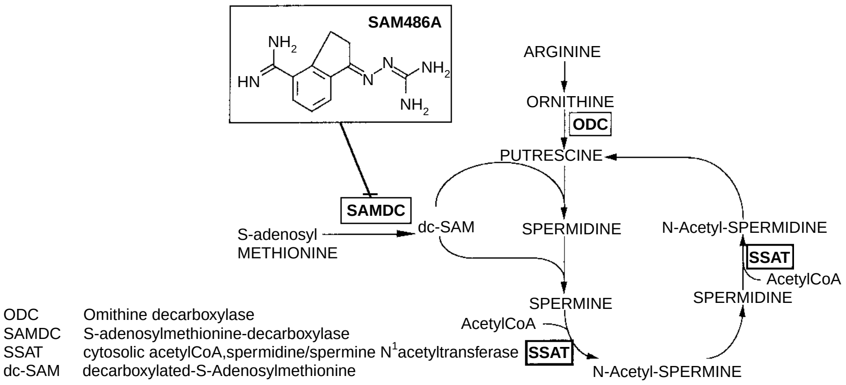

**Figure 1** Polyamine biosynthetic pathway rats and dogs was multi-exponential with extensive distribution outside the plasma compartment, and a high uptake into the liver and salivary glands was seen. SAM 486A was hardly metabolized and predominantly renally excreted. Furthermore, as compared to the plasma elimination profile, a marked accumulation of the drug was seen in tumours of nude mice subcutaneously transplanted with the human HCT-116 tumour cell line (data on file).

Three clinical phase I trials have been initiated to characterize the tolerability, safety and maximum tolerated dose of SAM486A evaluating three different schedules of administration. In addition to the xenograft data described above, the observation that SAMDC activity recovered very rapidly after withdrawal of SAM486A suggests that a continuous exposure of the drug may be desirable to achieve an optimal control of tumour growth. Accordingly, two of the trials investigated regimens designed to achieve continuous drug exposure: a daily infusion over 5 consecutive days every 3 weeks (Siu et al, 1998) and a 120 h continuous intravenous schedule given every 4 weeks as reported in the present study, of which preliminary results were presented at the 1998 ASCO meeting (Paridaens et al, 1998). Based on the clinical experience with MGBG where a weekly administration turned out to be better tolerated (Warrell and Burchenal, 1983), the third study investigated a weekly administration schedule (Greim et al, 1998). Results from this study and the two parallel phase I studies will be used as the basis for dosage and schedule selection and for the safety evaluation of SAM486A in the future clinical programme.

### PATIENTS AND METHODS

### Patients

Patients aged 18 or more with any type of histologically confirmed solid tumour (including lymphoma), which was refractory to conventional therapy or for which no standard therapy existed, were eligible. In addition, patients needed to have a WHO performance status of 0–2, a life expectancy of ≥3 months, and normal hepatic, renal and cardiac function. Informed consent was obtained in accordance to local regulatory requirements, and with the Declaration of Helsinki.

### Treatment and dose escalation

SAM486A (Novartis Pharma AG) was supplied as vials containing 10 or 50 mg of freeze-dried, light yellow powder for reconstitution in a dextrose solution. The drug was diluted in 1000 ml 5% dextrose for 1 day of treatment. Treatment was given to cohorts of patients (minimum size 2–3 patients) as a 120 h continuous infusion every 4 weeks. A maximum of six cycles was foreseen. Dosing began at 3 mg $m^{-2}$ $cycle^{-1}$, calculated as one third of the human equivalent of the no-adverse-effect level in the rat, i.e. 0.06 mg $kg^{-1}$ $day^{-1}$ (unpublished). Dose escalation was performed in increments of 100–50% if none or minor grade 1 toxicity (NIH common toxicity criteria, CTC) was observed, and by 33–22% increments if grade 2 or higher toxicity occurred. In case of grade 2 or greater toxicity cohort size was expanded to six patients. Dose escalation was continued until dose-limiting toxicity was observed. Escalation decisions were made jointly between investigators and sponsor following assessment of safety and pharmacokinetic data at each cohort.

### Dose-limiting toxicity and maximum tolerated dose

A nontolerated dose level was defined as a dose level on which, among all treated patients (maximum six), either at least three had grade 3 haematological toxicity, or at least two had grade 4 haematological toxicity; or at least three had grade 3 non-haematological toxicity; or at least one patient had grade 4 non-haematological toxicity. The maximum tolerated dose (MTD), and hence, recommended dose for Phase II trials, was defined as the highest dose not associated with dose-limiting toxicity as described above.

### SAM486A/5FU combination

Patients who received more than two courses were to have 5-Fluorouracil (5FU) added to their treatment from the third cycle onwards, based on preclinical data which showed additive antiproliferative action when these two drugs were given together. They received five daily bolus injections of 5FU at a daily dose of 500 mg $m^{-2}$ $day^{-1}$, administered in combination with SAM486A starting at cycle three, and continuing as long as the patient remained in the trial.

### Evaluation of safety and efficacy

Safety was assessed by monitoring of vital signs and ECG daily during infusion, and weekly thereafter. Haematological and biochemical parameters were routinely monitored weekly. Physical examination was performed weekly. LVEF was monitored every

© 2000 Cancer Research Campaign second cycle or at discontinuation. Monitoring of adverse events was continuous during the patient's participation in the trial.

Efficacy was measured by assessment of overall tumour response using UICC criteria. Additionally biological (tumour) markers were measured when appropriate. Fluorodeoxy glucose Positron emission tomography (FDG-PET) was performed in two patients.

### Pharmacokinetic sampling and analysis

SAM486A was measured in plasma and urine using a standardized methodology previously described (Degen and Zbinden, 1996). During the first cycle, all patients underwent pharmacokinetic sampling; those who received a third cycle of treatment with the addition of 5FU had repeated pharmacokinetics during that cycle. Venous blood samples of 5 ml were collected in lithium or sodium heparin coated tubes prior to start of the infusion (0 h), at day 4, then 5, 15, 30 min, 1, 2, 4, 6, 8, 12 and 24 h after the end of the infusion. During the first cycle (and third when 5FU was given), urine was also collected in three 8-h samples on day 1, following the administration of SAM486A. For each fraction, the total volume was recorded and a 10 ml aliquot was stored at –20°C until pharmacokinetic analysis.

The pharmacokinetic parameters $C_{max}$, $AUC_{(0\text{-}\infty)}$ and $t_{1/2}$ were determined by non-compartmental analysis using WinNonlin Professional (Version 1.5) software (Scientific Consulting, Inc). Descriptive statistics of the pharmacokinetic parameters were calculated and included mean, SD, median and range (minimum and maximum). The data was also analysed by linear regression.

### Pharmacodynamic sampling and analysis

Blood samples were collected during the first treatment cycle for measurement of polyamines and SAMDC activity. Blood samples (10 ml) were taken at baseline, 72 h and 120 h after the start of SAM486A infusion, and at days 7–8, 14, 21 and 28 after the start of treatment. Polyamine pools and SAMDC activity were measured in leukocytes using a published methodology (van den Berg et al, 1987). Analysis was descriptive only.

### FDG-PET scans

2-{$^{18}$F} fluoro-2-deoxy D-glucose PET was to be performed at University Hospital, Groningen, and University Hospital, Leuven, on consenting patients receiving the recommended dose for Phase II trials. Patients were scanned at baseline, not more than 72 h before the start of the CGP 48664 infusion, and 2–3 days following the first infusion.

{$^{18}$F}-FDG was synthesized under strict radiopharmaceutical controls using the method of Hamacher et al (1986). Statistical analysis was performed using standardized uptake values (Woodword et al, 1975) and Patlak analysis (Patlak et al, 1983).

### RESULTS

### Patient characteristics

From 7 December 1995 to 31 march 1998, 39 patients were enrolled into the study from two centres. All were caucasian, 26 males and 13 females. Only four patients (10%) had not received prior anticancer therapy. Patient characteristics are described in Table 1.

### Dose escalation and MTD of SAM486A monotherapy

Ten cohorts of 2–6 patients were studied at increasing dose levels ranging from 3–700 mg m$^{-2}$ cycle$^{-1}$. Nine patients had only one cycle of treatment, 21 (54%) had two, and three patients in each case had 3, 4 and 6 cycles. Dose escalation started at 3 mg m$^{-2}$ cycle$^{-1}$ and increased in 100–75% steps to 700 mg m$^{-2}$ cycle$^{-1}$. From this dose level two dose de-escalations to 550 mg m$^{-2}$ cycle$^{-1}$, then back to 400 mg m$^{-2}$ cycle$^{-1}$ were performed due to appearance of dose-limiting granulocytopenia at the top two dose levels. The MTD of SAM486A was defined as 400 mg m$^{-2}$ cycle$^{-1}$. The number of patients and cumulative dose per dose level is shown in Table 2.

### Safety

### *Haematological toxicity*

Granulocytopenia was the primary toxicity observed as a result of SAM486A administration. None of the two patients treated at 400 mg m$^{-2}$ cycle$^{-1}$ during the dose-escalation part of the study experienced haematological toxicity. However, some evidence of haematological toxicity was observed at this level among the four patients subsequently entered in this cohort during the dose de-escalation part of the study (one patient experienced transient grade 3 granulocytopenia, another patient grade 1 granulocytopenia). Of the six patients enrolled to receive a dose of 550 mg m$^{-2}$ cycle$^{-1}$, four experienced grade 3 or 4 granulocytopenia, and three of these were grade 4. At the highest dose level tested (700 mg m$^{-2}$ cycle$^{-1}$) three out of five patients experienced grade 4 granulocytopenia. The median day of granulocyte nadir was day 21 of the cycle. Fever associated with granulocytopenia was noted in three cases. Spontaneous recovery was observed, usually within 1 week of nadir and without administration of growth factors, although antibiotics were administered in two of these cases.

**Table 1** Patient characteristics

| | |
|---|---|
| Sex | |
| Male | 26 pts |
| Female | 13 pts |
| Median age (range) | 55 years (22–78) |
| Performance status at baseline | |
| 0 | 15 pts |
| 1 | 19 pts |
| 2 | 5 pts |
| Primary diagnosis | |
| Malignant melanoma | 7 pts |
| Pulmonary carcinoma | 4 pts |
| Renal carcinoma | 4 pts |
| Breast carcinoma | 4 pts |
| Colorectal carcinoma | 4 pts |
| Sarcoma | 3 pts |
| Adenocarcinoma of unknown primary site | 3 pts |
| Other | 10 pts |
| Prior anticancer therapy | |
| none | 4 pts |
| Surgery | 24 pts |
| Chemotherapy | 34 pts |
| Radiotherapy | 22 pts |
| Immunotherapy | 4 pts |
| Hormonal therapy | 10 pts |

 © 2000 Cancer Research Campaign

**Table 2** Dose escalation scheme and cumulative dose by dose level

| Dose level | 1 | 2 | 3 | 4 | 5 | 6 | 7 | 8 | 9 | 10 |
|---|---|---|---|---|---|---|---|---|---|---|
| Dose intended mg $m^{-2}$ $cycle^{-1}$ | 3 | 6 | 12 | 24 | 48 | 96 | 200 | 400 | 700 | 550 |
| Number of patients | 3 | 4 | 3 | 3 | 4 | 3 | 2 | 6 | 5 | 6 |
| Number of patients receiving 5FU | | 2 | 3 | | 1 | | | | | |
| Number of cycles given (including cycles with combination SAM486A/5FU) | 6 | 15 | 10 | 6 | 10 | 6 | 8 | 10 | 13 | 6 |
| Cumulative dose (mg) | | | | | | | | | | |
| Median | 11.3 | 40.5 | 72 | 82 | 188.75 | 330 | 1535 | 1154 | 1210 | 2113.25 |
| Sum | 31 | 157.8 | 229.8 | 242.5 | 815.5 | 985.5 | 3070 | 7465 | 7230 | 13314.5 |

There is a clear relationship between dose and incidence of haematological toxicity, as shown in Table 3. Thrombocytopenia was observed in five patients. In three of these patients granulocytopenia and leukopenia were also observed. Anaemia was noted in 54% of patients. However, grade 3–4 anaemia was noted only in four patients and represented worsening from grade 1 or 2 anaemia present at baseline in all cases.

### Non-haematological toxicity

The most common non-haematological adverse events which were considered related to SAM486A administration were nausea (26%), vomiting (21%), fatigue (26%) and anorexia (13%). Drug-related nausea was present throughout the treatment cycle, whereas fatigue, anorexia and vomiting were reported more frequently after the infusion (Table 4). Drug-related nausea occurring during the infusion was observed only in the three highest dose groups, whereas post-infusion nausea was also observed at lower dose levels. When anti-emetic therapy was used it was found to be effective in controlling these symptoms. Nausea and vomiting were of mild to moderate severity, as were the vast majority of the adverse events observed. Grade 3 fatigue was observed in only two patients (one at 400 mg $m^{-2}$ $cycle^{-1}$ and another at 700 mg $m^{-2}$ $cycle^{-1}$), grade 3 anorexia was observed in one patient at 550 mg $m^{-2}$ $cycle^{-1}$.

Grade 2–4 abnormalities in one or more liver function tests, particularly in alkaline phosphatase, gamma GT and LDH, were observed in 20/39 (51%) patients. This finding is tempered by the observation that some abnormality (grade 1 abnormality or greater) in one or more liver function tests was observed at baseline in 30/39 (77%) patients, and in 20/39 (51%), two or more parameters were already abnormal at baseline. Of the patients with abnormalities in one or more liver function tests at baseline, 15 entered the study with documented tumour involvement in the liver. Hence, the overall interpretation of liver function test abnormalities is confounded by the disease status of the patient population enrolled onto the study. Overall, there is little evidence for increased incidence of grade 2 or worse abnormalities in liver function tests with increased doses of SAM486A. There were no significant drug-related changes in electrolytes or in renal function, nor in LVEF. Only one patient experienced an episode of atrial fibrillation after cycle 2. This patient had a prior history of atrial fibrillation and the event was not considered related to the study drug.

### Clinical efficacy

No complete or partial responses were recorded in any of the 39 patients. Three patients completed the full six cycles of treatment. Of these, two received the combination 5FU/SAM486A from cycle 3 onwards. Five patients recorded a best response of stable disease. Of these, three received the combination 5FU/SAM486A from cycle 3 onwards (see below).

**Table 3** Haematological toxicity by dose level (brackets indicate occurrence with combination SAM486A/5FU)

| | SAM486A intended dose level (mg $m^{-2}$ $cycle^{-1}$) | | | | | | | | | | |
|---|---|---|---|---|---|---|---|---|---|---|---|
| | 3 | 6 | 12 | 24 | 48 | 96 | 200 | 400 | 550 | 700 | Total (%) |
| Total no. of patients studied | 3 | 4 | 3 | 3 | 4 | 3 | 2 | 6 | 6 | 5 | 39 (100) |
| No of patients receiving 5FU | – | 2 | 3 | – | 1 | – | – | – | – | – | – |
| Grade 3–4 | | | | | | | | | | | |
| Leukocytes (WBC) | – | (1) | – | – | – | – | – | – | 3 | 3 | 7 (18) |
| Granulocytes (AGC) | – | (1) | – | – | – | – | – | 1 | 4 | 3 | 9 (23) |
| Thrombocytes | – | 1 | – | – | – | – | – | – | 1 | – | 2 (5) |
| Haemoglobin | – | – | 1 | 1 | – | – | – | – | 1 | 1 | 4 (10) |
| Grade 1–4 | | | | | | | | | | | |
| Leukocytes (WBC) | – | (1) | (1) | – | – | – | 1 | 2 | 5 | 4 | 14 (36) |
| Granulocytes (AGC) | – | (1) | (1) | – | – | – | – | 2 | 4 | 4 | 12 (31) |
| Thrombocytes (platelets) | – | 1 | (1) | – | – | – | – | – | 1 | 2 | 5 (10) |
| Haemoglobin | 2 | 2 | 2 | 3 | 2 | 1 | – | 3 | 4 | 2 | 21 (54) |

*© 2000 Cancer Research Campaign* 

**Table 4** Non-haematological toxicity (considered related to SAM486A) by dose level (brackets indicate occurrence with combination SAM486A/5FU)

| | SAM486A intended dose level (mg $m^{-2}$ $cycle^{-1}$) | | | | | | | | | | |
|---|---|---|---|---|---|---|---|---|---|---|---|
| | 3 | 6 | 12 | 24 | 48 | 96 | 200 | 400 | 550 | 700 | Total (%) |
| Total No. of patients studied | 3 | 4 | 3 | 3 | 4 | 3 | 2 | 6 | 6 | 5 | 39 (100) |
| No. of patients receiving 5FU | – | 2 | 3 | – | 1 | – | – | – | – | – | |
| Grade 3–4 | | | | | | | | | | | |
| During infusion (day 1–5) | | | | | | | | | | | |
| Condition aggravated | – | – | – | – | – | – | – | – | – | 1 | 1 (3) |
| Asthenia | – | – | – | – | – | – | – | – | 1 | – | 1 (3) |
| Vesicular rash | – | – | – | – | – | – | – | – | – | 1 | 1 (3) |
| Vomiting | – | – | – | – | – | – | – | – | – | 1 | 1 (3) |
| After infusion (day 6–28) | | | | | | | | | | | |
| Fatigue | – | – | – | – | – | – | – | 1 | – | 1 | 2 (5) |
| Condition aggravated | – | – | – | – | – | – | – | – | 1 | – | 1 (3) |
| Mucositis nos | – | – | (1) | – | – | – | – | – | – | – | 1 (3) |
| Atrial fibrillation | – | – | – | – | – | – | – | – | 1 | – | 1 (3) |
| Anorexia | – | – | – | – | – | – | – | – | 1 | – | 1 (3) |
| Dementia | – | – | – | – | – | – | – | – | – | 1 | 1 (3) |
| Somnolence | – | – | – | – | – | – | – | – | – | 1 | 1 (3) |
| Dyspnea | – | – | – | – | – | – | – | – | – | 1 | 1 (3) |
| Vesicular rash | – | – | – | – | – | – | – | – | – | 1 | 1 (3) |
| Grade 1–4 (most common > 10%) | | | | | | | | | | | |
| During infusion (days 1–5) | | | | | | | | | | | |
| Nausea | – | – | – | – | – | – | – | 1 | 2 | 2 | 5 (13) |
| After infusion (days 6–28) | | | | | | | | | | | |
| Nausea | – | 1 | – | 1 | (1) | 1 | – | 1 | 3 | 2 | 10 (26) |
| Fatigue | 1 | 1 | 1 | 1 | – | – | – | 2 | 2 | 2 | 10 (26) |
| Vomiting | – | – | – | 1 | – | 1 | – | – | 3 | 3 | 8 (21) |
| Anorexia | 1 | – | – | – | – | – | – | 3 | 1 | – | 5 (13) |

Measurements of tumour markers were evaluable in only six patients (CEA in four patients, CA 15.3 in two patients). No trends towards decreases in post-baseline values were observed.

FDG-Positron Emission Tomography (PET) was performed in only three patients at dose level 400 mg $m^{-2}$ $cycle^{-1}$, In two patients, PET scan following the first 5-day infusion was performed (2 days following end of infusion). Results were suggestive of stable disease at best (not shown). Both patients discontinued due to disease progression after 1–2 cycles of SAM486A treatment, respectively. The results reflect the refractory nature of disease in these patients and possibly suggest the need to further optimize the time at which the post-treatment scan is performed.

### Clinical experience with the combination of SAM486A and 5FU

Per protocol, six patients received combined treatment with SAM486A and 5FU from cycle 3 (two at a dose of SAM486A of 6 mg $m^{-2}$ $cycle^{-1}$, three at 12 mg $m^{-2}$ $cycle^{-1}$ and one at 48 mg $m^{-2}$ $cycle^{-1}$). Two of them experienced a severe toxicity. In one patient at 6 mg $m^{-2}$ $cycle^{-1}$ SAM486A, a grade 4 granulocytopenia was observed on day 20 of the first combination cycle. Following 5FU dose reduction to 300 mg $m^{-2}$ $day^{-1}$ for 5 consecutive days (vs the original dose of 500 mg $m^{-2}$ $day^{-1}$) the patient received a further three cycles of combination treatment without recurrence of myelosuppression. A second patient receiving 12 mg $m^{-2}$ $cycle^{-1}$ developed a grade 3 mucositis, which resolved by the end of the cycle and did not recur upon rechallenge with a reduced dose of 5FU (300 mg $m^{-2}$ $day^{-1}$) during cycle 4. Consequently, as an interaction between both drugs could not be ruled out at this early stage, the study of the combination was suspended (blood levels of SAM486A were inconclusive, all below detection limits at these low-dose levels).

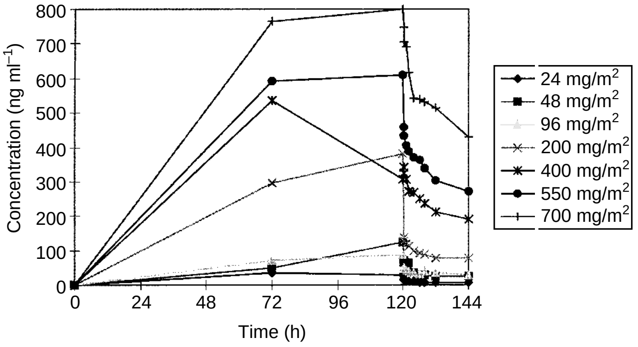

**Figure 2** Mean plasma drug concentration–time profiles for dose levels 24–700 mg $m^{-2}$ $cycle^{-1}$

### Pharmacokinetics and pharmacodynamics

The first 10 patients included at lowest dose levels had plasma SAM486A concentrations below the limit of quantitation during significant portions of the sampling interval. Mean plasma drug concentration – time profiles for dose levels 24–700 mg $m^{-2}$ $cycle^{-1}$ are shown in Figure 2. Although there is substantial variability, both AUC and $C_{max}$ are linearly related to dose, as illustrated in Figure 3. In both cases the regression line passes close to the origin (intercept is low). The dose per cycle explained 68% and 70% of the AUC and $C_{max}$ variabilities, respectively, as shown by the R2 statistic. Mean terminal elimination half-life, in contrast, did not appear to be related to dose. For all patients included in the analysis the $t_{1/2}$ was 48.6 h, with a standard deviation of 23.1 h. Approximately 3% of the total drug administered was excreted in the urine on day 1.

 *© 2000 Cancer Research Campaign*

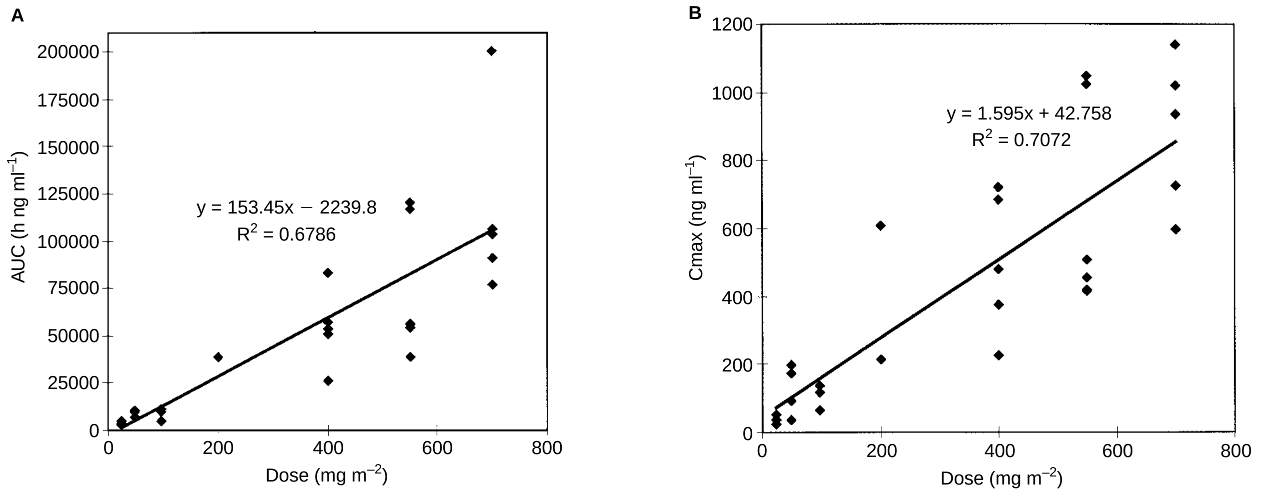

**Figure 3** Correlations between dose and AUC (**A**) and dose and $C_{max}$ (**B**)

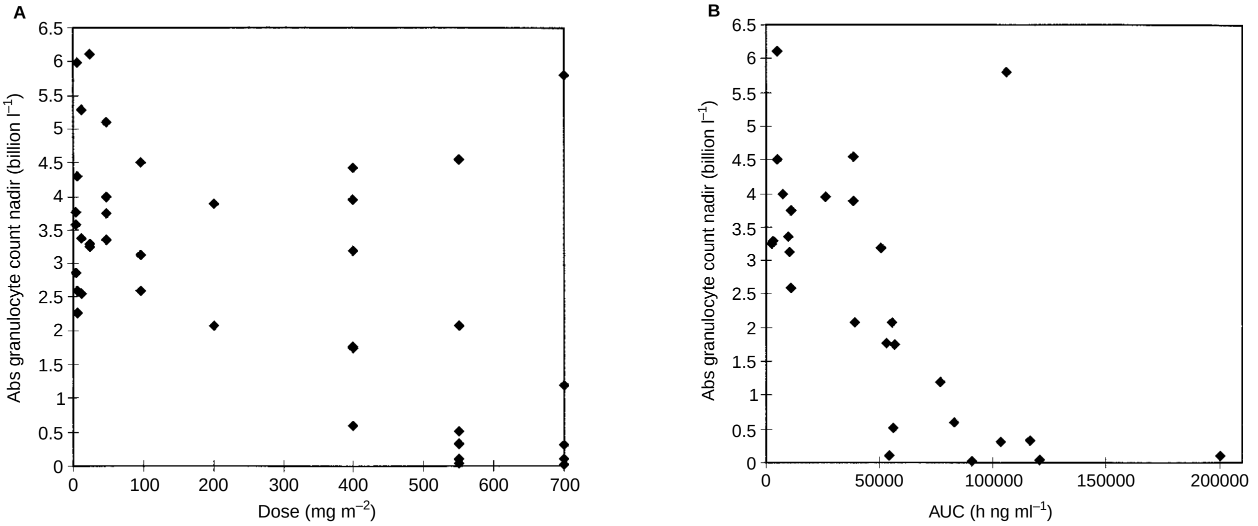

**Figure 4** Correlations between granulocytopenia and dose (**A**) and granulocytopenia and AUC (**B**)

Plasma levels observed in this study far exceeded the $IC_{50}$ of 0.0047 μM (1.1 ng ml$^{-1}$) required in in vitro experiments for inhibition of SAMDC, suggesting that biologically relevant levels were achievable at a dose of 200 mg m$^{-2}$ cycle$^{-1}$ but were more sustained at doses of 400 mg m$^{-2}$ cycle$^{-1}$ and above.

Correlative analysis between pharmacokinetic parameters and adverse events showed that doses of 550 and 700 mg m$^{-2}$ cycle$^{-1}$ and, most strikingly, AUC > 60 000 h ng ml$^{-1}$ correlated with grade 3 or 4 granulocytopenia (correlation coefficient 0.5814 and 0.6931, respectively, Figure 4. For $C_{max}$, a trend is apparent toward greater incidence of granulocytopenia at higher $C_{max}$ (correlation coefficient 0.6794). In contrast, no correlations between pharmacokinetic parameters and the adverse events nausea and vomiting, fatigue and anorexia were found.

With respect to pharmacodynamic assessments, the polymines putrescine, spermidine and spermine and SAMDC activity were measured in peripheral blood leukocytes. The data suggest a trend towards increased levels of putrescine following the treatment period at doses of 400 mg m$^{-2}$ cycle$^{-1}$ and above in 10 of the 11 patients evaluable (in contrast, such an increase was seen in six of the 12 patients evaluable treated at doses of 3–200 mg m$^{-2}$ cycle$^{-1}$). However, the interpatient variability was marked in terms of both the magnitude of the increase and its time course, and furthermore the expected concomitant decrease in the higher polyamines spermidine and spermine could not be detected.

SAMDC activity showed a marginal trend towards an increase in some patients (nine of 18 patients and eight of 13 patients evaluable, and treated at doses up to 200 mg m$^{-2}$ cycle$^{-1}$ and at doses of 400 mg m$^{-2}$ cycle$^{-1}$ and above, respectively), followed by a decrease in values during the 28-day treatment cycle. There was no clear-cut dose–response relationship. Such an increase is consistent with drug action and is thought to be due to an artefactual stabilization of the SAMDC enzyme. However, again, interpatient variability makes further interpretation of these observations difficult.

© 2000 Cancer Research Campaign

### DISCUSSION

The primary objectives of this phase I trial, namely to evaluate safety and tolerability of SAM486A given as a 5-day continuous infusion in a population of patients with advanced cancer, and to determine the MTD, have been fulfilled. SAM486A is well tolerated up to and including a dose of 400 mg $m^{-2}$ $cycle^{-1}$, and cycles can be repeated every 4 weeks without evidence of cumulative toxicity or necessity for cycle interruption. Dose-limiting toxicity is observed at doses of 550 mg $m^{-2}$ $cycle^{-1}$ and 700 mg $m^{-2}$ $cycle^{-1}$ and consists of reversible, uncomplicated granulocytopenia. This is clearly dose-dependent, being observed almost exclusively within the top three dose levels and becoming intolerable in the top two dose levels explored. The granulocytopenia is relatively late in onset with a median nadir occurring on cycle day 21 (given the caveat of weekly sampling only in most patients). Granulocytopenia was uncomplicated by fever in most cases and recovery was usually observed within 7 days. The MTD of 400 mg $m^{-2}$ $cycle^{-1}$ induced grade 3 granulocytopenia in one of six patients: this was manageable and furthermore not dose-limiting as defined by protocol. However it must be recognized that mild granulocytopenia may well be expected in some patients when SAM486A is given at this dose and schedule.

Mild to moderate nausea and vomiting was also reported throughout the cycle, occurring in 15% during infusion, and in 31% thereafter. The causal relationship with therapy was not always evident and could, at least in some cases, merely be ascribed to the disease. Anti-emetic therapy was given on a number of occasions and was effective in the control of potential further emetic episodes. However, given the mild nature of most cases, prophylactic anti-emetic therapy is not likely to be indicated based upon these observations. Preclinical animal testing did not indicate that SAM486A may induce emesis.

Fatigue was also observed as a frequent event, occurring in 51% of all patients after the infusion. This effect was considered by the clinicians as possibly due to study drug in about half (26%) of these patients. The general intolerability of doses 550 and 700 mg $m^{-2}$ $cycle^{-1}$ is also reflected by the observation that eight of the 11 patients treated at these dose levels experienced some worsening in performance status.

On the basis of encouraging preclinical data, patients proceeding to a third cycle of SAM486A treatment were offered daily bolus 5FU (at a dose of 500 mg $m^{-2}$ $day^{-1}$ for 5 consecutive days) in combination with the continuous infusion SAM486A. However, this combination was explored only at low-dose levels of SAM486A, as grade 4 granulocytopenia and grade 3 mucositis were observed at the second and third SAM486A dose levels, respectively, leading to the suspension of this combination in this protocol. These toxicities, particularly mucositis, are known side-effects of 5 FU treatment but have also been observed in patients treated with MGBG, of which SAM486A is an analog. Although no mucositis was reported with single-agent SAM486A at any dose, and no granulocytopenia with single-agent SAM486A at doses where the combination was given, an additive effect cannot be ruled out. The question of potential interaction between SAM486A and 5 FU needs to be addressed in a study designed specifically to address this issue, and further development in indications where 5FU is commonly used (e.g. colorectal cancer) is warranted.

SAM486A undergoes multiphasic elimination from the plasma, characterized by an initial rapid decline followed by a gradual prolonged decline. The mean terminal elimination half-life of SAM486A on this schedule is approximately 2 days and $C_{max}$ and AUC are linearly related to dose. Furthermore, the plasma levels achieved at doses ≥ 200 mg $m^{-2}$ $cycle^{-1}$ exceeded by far the $IC_{50}$ for in vitro inhibition of SAMDC (0.0047 μM). This finding suggests that biologically relevant levels of SAM486 are achievable in man, but this will need to be confirmed by direct measurement of drug level in tumour biopsies from treated patients. Of interest, in the study by Siu et al (1998) investigating a daily regimen over 5 consecutive days, a potent inhibition of SAMDC (reflected by a marked decrease in decarboxylated S-adenosyl methionine) was shown in one patient with metastatic melanoma who had pre- and post-treatment tumour biopsies (Porter et al, 1998) The disposition of SAM486A in rats and dogs was characterized by rapid clearance from blood and plasma, an extensive distribution throughout the body, accumulation in various tissues, particularly in the liver, incomplete (predominantly renal) excretion and a low degree of metabolism. Elimination from tissues was markedly slower than from blood and plasma. This relatively long terminal elimination half-life is consistent with the preclinical findings. The linearity of the pharmacokinetic parameters $C_{max}$ and AUC with dose implies that there are no saturable, inhibited or induced processes involved with the distribution and elimination of SAM486A. This would facilitate the adjustment of doses to achieve targeted plasma drug levels or exposure (AUC).

The dose-limiting toxicity, granulocytopenia, is associated with doses greater than 400 mg $ml^{-2}$ $cycle^{-1}$ and AUC > 60 000 h ng $m^{-1}$. This clear relationship suggests that PK monitoring might be helpful in adjusting the dose in response to incidences of granulocytopenia. Correlative analysis suggests that there is no correlation of PK parameters with incidence and severity of nausea and vomiting, fatigue and anorexia. This suggests that these adverse events may not be related to exposure to SAM486A (i.e. no dose–event relationship).

No evidence of antitumour activity in the form of objective tumour regression was observed in this heterogeneous and heavily pretreated population of patients with advanced cancer. However, stable disease was recorded as best response in five patients. Repeated positron emission topography (PET) scanning using fluoro-deoxy glucose was performed on only two patients treated at the highest dose levels following the first 5-day infusion. The observed results were suggestive of disease stabilization at best. Despite these inconclusive results, the technique per se remains a powerful technology for the detection of subclinical drug activity by investigating tumour metabolism. Thus, with some refinement, it would seem worthwhile continuing to explore this technique as part of future clinical studies, in a more favourable population of patients likely to respond to treatment, perhaps with modification of the scanning protocol to investigate later time-points following end of infusion.

Investigation of polyamine pools in peripheral blood leukocytes was hampered by a high interpatient variability at any dose of SAM486A. Changes were inconsistent and not uniformly dose-related, with some possible trends consistent with the proposed mechanism of action in selected polyamine parameters. As shown in experimental models, the dependence of cell survival on endogenously synthesized polyamines in a given tissue appears to be a function of its proliferative rate (Hassels et al, 1989). Thus, in view of the inconsistency between perceived trends, and because circulating leucocytes may be irrelevant as surrogate pharmacodynamic

 *© 2000 Cancer Research Campaign*

marker of tumour response, it does not appear that polyamine levels in peripheral blood leukocytes is a suitable means to monitor pharmacodynamic action of SAM486A.

In conclusion, the recommended dose for further phase II–III studies using this schedule is 400 mg $m^{-2}$ $cycle^{-1}$, to be repeated every 4 weeks. Mild reversible haematotoxicity (leucopenia and thrombopenia), fatigue, nausea and vomiting may be observed, the latter responding well to standard anti-emetic treatments. At higher dosages, severe (grade 3 or 4) neutropenia becomes the dose-limiting toxicity. Given the good tolerability profile of SAM486A and the promising antitumour activity observed in preclinical models, single-agent testing in a phase II setting is clearly warranted. The optimal dosing and scheduling of a SAM486A/5-FU combination requires further phase I investigation, but the observation of disease stabilization in patients receiving this combination points to the need for evaluation of this combination in indications where 5FU is commonly used, such as advanced colorectal cancer.

### REFERENCES

Degen PH and Zbinden P (1996) Automated quantitative determination of a new polyamine biosynthesis inhibitor (CGP 48664) and a potential metabolite in human and in animal plasma by high-performance liquid chromatography. *J Chromatogr* **681**: 339–345

Delworth MG, Nishioka K, Pettaway C, Gutman M, Killion JJ, von Eschenbach AC and Fidler IJ (1997) Systemic administration of 4-amidinoindanon-1-(2′-amidino)/hydrazone, a new inhibitor of S-adenosylmethionine decarboxylase, produces cytostasis of human prostate cancer in athymic nude mice. *Int J Oncology* **6**: 293–299

Dorhout B, Te Velde RJ, Ferwerda H, Kingma AW, De Hoog E and Muskiet FAJ (1995). In-vivo growth inhibition of L1210 leukemia by 4-amidinoindan-1-one 2′-amidinohydrazone (CGP 48664A), a new inhibitor of S-adenosylmethionine decarboxylase. *Int J Cancer* **61**: 214–217

Greim G, Bruntsch U, Eskens F, Höppener F, Barbet NC, Choi L, Hanauske A-R and Verweij J (1998) Phase I and pharmacologic study of CGP 48664, a SAMDC inhibitor given once weekly ×4 in patients with solid tumors. *Proc ASCO* **17**: 232

Gschaidmeier H, O'Reilly T, Stanek J and Mett H (1999) Additive/synergistic antitumor activity of SAM486A (CGP 48664) combined with cytotoxic drugs in tissue culture and in nude mice. European Concerted Action (COST) Subgroup meeting 917: Bad Nauheim, Germany

Gutman M, Beltran PJ, Fan D, Delworth M, Singh RK, Wilson M and Fidler IJ (1995) Treatment of nude mice with 4-amidinoindanon-1-(2′-amidino) hydrazone, a new S-adenosylmethionine decarboxylase inhibitor, delays growth and inhibits metastasis of human melanoma cells. *Melanoma Research* **5**: 147–155

Hamacher K, Coenen HH and Stocklin G (1986) Efficient stereospecific synthesis of no-carrier-added 2-[18F]-fluoro-2-deoxy-D-glucose using aminopolyether supported nucleophilic substitution. *J Nucl Med* **27**: 235–238

Hassels J, Kingma AW, Ferwerda H, Keij J, van den Berg GA and Muskiet FAJ (1989) Microbial flora in the gastrointestinal tract abolishes cytostatic effects of α-difluoromethylornithine in vivo. *Int J Cancer* **43**: 1155–1164

Manni A, Badger B, Wechter R, Kunselman S, Rossini A and Demers L (1995) Biochemical and growth-modulatory effects of the new S-adenosyl methionine decarboxylase inhibitor CGP 48664 in malignant and immortalized normal breast epithelial cells in culture. *Int J Cancer* **62**: 486–491

McCann PP and Pegg AE (1992) Ornithine decarboxylase as an enzyme target for therapy. *Pharmacol Ther* **54**: 195–215

Mi Z, Kramer DL, Miller JT, Bergeron RJ, Bernacki R and Porter CW (1998) Human prostatic carcinoma cell lines display altered regulation of polyamine transport in response to polyamine analogs and inhibitors. *The Prostate* **34**: 51–60

O'Reilly T, Cozens R and Mett H (2000) Evaluation of the antitumor activity of the S-adenosylmethionine decarboxylase (SAMDC) inhibitor SAM486 alone and in combination with conventional cytotoxic agents. American Association for Cancer Research 91st meeting, abstract submitted for publication.

Paridaens R, Uges DRA, Barbet N, Seeghers M, van der Graaf WTA, Lassus M, Groen HJM, Dumez H, Muskiet F, Man A, van Oosterom AT and De Vries EGE (1998) Phase I dose escalation and pharmacokinetic study of CGP 48664, a new S-adenosyl-methionine decarboxylase inhibitor, administered in continuous infusion over 5 days in cancer patients with solid tumors. *Proc ASCO* **17**: 190

Patlak CS, Blasberg RG and Fenstermacher JD (1983) Graphical evaluation of blood-to-brain transfer constants from multiple-time uptake data. *J Cereb Blood Flow Metab* **3**: 1–7

Pegg AE and McCann PP (1992) S-adenosylmethionine decarboxylase as an enzyme target fortherapy. *Pharmacol Ther* **56**: 359–377

Porter CW, Regenass U and Bergeron RJ (1992) Polyamine inhibitors and analogues as potential anticanceragents. In: *Polyamines in the gastrointestinal tract*. Falk Symposium 62, Dowling RH, Fölsch UR, Löser R (eds) pp301–322, Kluwer Academic Publishers, Dordrecht

Porter CW, Siu L, Kramer DL, Mett H, Barbet N, Linnartz R and Eckhardt SG (1998) Pharmacologic confirmation of CGP-48664 as an inhibitor of the polyamine biosynthetic enzyme S-Adenosylmethionine Decarboxylase (SAMDC) in an advanced melanoma patient. 10th NCI-EORTC symposium on new drugs in cancer therapy, June 16–19, 1998 Amsterdam, p. 128, abstr. no. 491

Regenass U, Mett H, Stanek J, Mueller M, Kramer D and Porter CW (1994) CGP 48664, a new S-adenosyl methionine decarboxylase inhibitor with broad spectrum antiproliferative and antitumor activity. *Cancer Res* **54**: 3210–3217

Siu LL, Rowinsky EK, Weiss GR, Hammond L, Kraynak M, Moczygemba J, Choi L, Barbet NC, Demoor C, Von Hoff DD and Eckhardt SG (1998) A Phase I and pharmacokinetic study of the polyamine biosynthesis inhibitor CGP 48664 in patients with advanced cancer. *Proc ASCO* **17**: 191

Svensson F, Mett H and Persson L (1997) CGP 48664 a potent and specific S-adenosyl-methionine decarboxylase inhibitor: effects on regulation and stability of the enzyme. *Biochemical Journal* **322**: 297–302

van den Berg GA, Kingman AW and Muskiet FAJ (1987) Determination of polyamines in human erythrocytes by capillary gas chromatography with nitrogen phosphorus detection. *J Chromatogr. Biomed. Appl* **415**: 27–34

Warrell RP and Burchenal JH (1983) Methylglyoxal-bis (guanylhydrazone) (methyl-GAG): current status and future prospects. *J Clin Onc* **1**: 52–65

Woodword HQ, Gigler RE and Freed B (1975) Expression of tissue isotope distribution. *J Nucl Med* **16**: 958–959

© 2000 Cancer Research Campaign

## References

[PDF_00864] Degen P. H., Zbinden P. (1996). Automated quantitative determination of a new polyamine biosynthesis inhibitor (CGP 48 664) and a potential metabolite in human and animal plasma by high-performance liquid chromatography.. J Chromatogr B Biomed Appl.

[PDF_00873] Dorhout B., Te Velde R. J., Ferwerda H., Kingma A. W., De Hoog E., Muskiet F. A. (1995). In vivo growth inhibition of L1210 leukemia by 4-amidinoindan-1-one 2'-amidinohydrazone (CGP 48664A), a new inhibitor of S-adenosylmethionine decarboxylase.. Int J Cancer.

[PDF_00884] Gutman M., Beltran P. J., Fan D., Delworth M. G., Singh R. K., Wilson M. R., Fidler I. J. (1995). Treatment of nude mice with 4-amidinoindan -1- one2 '- amidinohydrazone, a new S-adenosylmethionine decarboxylase inhibitor, delays growth and inhibits metastasis of human melanoma cells.. Melanoma Res.

[PDF_00888] Hamacher K., Coenen H. H., Stöcklin G. (1986). Efficient stereospecific synthesis of no-carrier-added 2-[18F]-fluoro-2-deoxy-D-glucose using aminopolyether supported nucleophilic substitution.. J Nucl Med.

[PDF_00891] Hessels J., Kingma A. W., Ferwerda H., Keij J., van den Berg G. A., Muskiet F. A. (1989). Microbial flora in the gastrointestinal tract abolishes cytostatic effects of alpha-difluoromethylornithine in vivo.. Int J Cancer.

[PDF_00894] Manni A., Badger B., Wechter R., Kunselman S., Rossini A., Demers L. (1995). Biochemical and growth-modulatory effects of the new S-adenosylmethionine decarboxylase inhibitor CGP 48664 in malignant and immortalized normal human breast epithelial cells in culture.. Int J Cancer.

[PDF_00898] McCann P. P., Pegg A. E. (1992). Ornithine decarboxylase as an enzyme target for therapy.. Pharmacol Ther.

[PDF_00900] Mi Z., Kramer D. L., Miller J. T., Bergeron R. J., Bernacki R., Porter C. W. (1998). Human prostatic carcinoma cell lines display altered regulation of polyamine transport in response to polyamine analogs and inhibitors.. Prostate.

[PDF_00914] Patlak C. S., Blasberg R. G., Fenstermacher J. D. (1983). Graphical evaluation of blood-to-brain transfer constants from multiple-time uptake data.. J Cereb Blood Flow Metab.

[PDF_00917] Pegg A. E., McCann P. P. (1992). S-adenosylmethionine decarboxylase as an enzyme target for therapy.. Pharmacol Ther.

[PDF_00929] Regenass U., Mett H., Stanek J., Mueller M., Kramer D., Porter C. W. (1994). CGP 48664, a new S-adenosylmethionine decarboxylase inhibitor with broad spectrum antiproliferative and antitumor activity.. Cancer Res.

[PDF_00937] Svensson F., Mett H., Persson L. (1997). CGP 48664, a potent and specific S-adenosylmethionine decarboxylase inhibitor: effects on regulation and stability of the enzyme.. Biochem J.

[PDF_00943] Warrell R. P., Burchenal J. H. (1983). Methylglyoxal-bis(guanylhydrazone) (Methyl-GAG): current status and future prospects.. J Clin Oncol.

[PDF_00945] Woodard H. Q., Bigler R. E., Freed B. (1975). Letter: Expression of tissue isotope distribution.. J Nucl Med.

[PDF_00940] van den Berg G. A., Kingma A. W., Muskiet F. A. (1987). Determination of polyamines in human erythrocytes by capillary gas chromatography with nitrogen-phosphorus detection.. J Chromatogr.

